# Methodological considerations for neonatal trials involving multiples: lessons from the bracelet study (bereavement and randomised controlled trials)

**DOI:** 10.1186/1745-6215-14-S1-O43

**Published:** 2013-11-29

**Authors:** Claire Snowdon, Peter Brocklehurst, Robert Tasker, Martin Ward Platt, Diana Elbourne

**Affiliations:** 1 London School of Hygiene & Tropical Medicine, London, UK; 2 National Perinatal Epidemiology Unit, University of Oxford, Oxford, UK; 3 Institute for Women’s Health, University College London, London, UK; 4 Department of Neurology, and Anaesthesia (Pediatrics), Harvard Medical School, Boston, USA; 5 Newcastle Neonatal Service, Royal Victoria Infirmary, Newcastle-upon-Tyne, UK

## 

Neonatal trials which include preterm babies often recruit multiples (twins or higher order births). For such trials, methodological decisions must be made regarding recruitment and randomisation of multiples. Enrolment can take place in complicated and challenging situations which are compounded if one or more babies die. In the BRACELET Study (Bereavement and Randomised Controlled Trials) (http://www.bracelet-study.org.uk), we conducted 30 interviews with 51 bereaved parents of babies entered into one of five neonatal intensive care trials, including 13 interviews with 22 parents of multiples, as well as 58 professionals (clinicians and/or trial team members). Parental interviews highlighted the array of circumstances which can exist for parents of multiples enrolled in trials. Issues discussed with professionals included:

• Excluding multiples and the impact upon statistical power, and generalisability.

• Randomisation policies

• o Individual randomisation (may receive same or different allocation)

• o ‘Group' randomisation (both/all to the same treatment)

• o Randomisation time-points (siblings may become eligible at the same or different times)

• Analysis of outcomes for multiples (presents issues of non-independence which need more complex statistical methods)

• Policies on feedback of trial results to parents (needs to take into account enrolment, allocation, and outcomes, including the death of one or more of baby in a family)

Including multiples in neonatal trials is important, but interviews from the BRACELET Study show the need to consider the complexity of the issues raised in the conduct of trials on both scientific and compassionate grounds.

